# Type and Volume of Milk Intake in Premature Infants <33 Weeks Gestational Age in the Neonatal Intensive Care Unit

**DOI:** 10.3390/children12040431

**Published:** 2025-03-29

**Authors:** Sudha Rani Narasimhan, Maricela Vallejo, Matthew Nudelman, Priya Jegatheesan

**Affiliations:** 1 Division of Neonatology, Department of Pediatrics, Santa Clara Valley Medical Center, San Jose, CA 95128, USA; matthew.nudelman@hhs.sccgov.org (M.N.); priya.jegatheesan@hhs.sccgov.org (P.J.); 2 Department of Pediatrics, Stanford University School of Medicine, Stanford, CA 94305, USA; 3 Department of Internal Medicine-Pediatrics, Medical College of Wisconsin Affiliated Hospitals, Milwaukee, WI 53226, USA; mvallejo@mcw.edu; 4 Department of Medicine, Section of Applied Clinical Informatics, University of Wisconsin School of Medicine and Public Health, Madison, WI 53726, USA

**Keywords:** breast milk, human milk, preterm feeding, breastfeeding

## Abstract

Background: Understanding the patterns of milk intake in the neonatal intensive care unit (NICU) will allow opportunities to intervene to improve mother’s milk supply. Objective: To quantify the type and volume of milk intake in premature infants throughout the NICU stay. Methods: This retrospective observational cohort study included infants born and admitted to the NICU at <33 weeks gestation from January 2014 to December 2017 who did not have contraindications for receiving mother’s own milk (MOM). Daily volume of MOM, pasteurized donor milk (PDM), and formula throughout the NICU stay were collected. Infants were categorized as exclusive human milk diet (EHM) if they consumed MOM and PDM or mixed diet if they consumed formula and MOM and/or PDM. Demographics, feeding outcomes, growth outcomes, and neonatal morbidities were collected. Results: Of 195 study infants, 133 (32%) received EHM. Cumulative volume and percent of MOM intake were greater in the EHM group compared to the mixed diet group. Age of first colostrum administration to infant was earlier in the EHM group than the mixed diet group (3.1 vs. 4.9, *p* = 0.013). By the second week of life, the EHM group received 100% of their feeds as MOM but the maximum MOM received in the mixed diet group was 63%. There was no difference in other feeding or neonatal outcomes between the groups. Conclusion: The EHM group received colostrum earlier than those who received a mixed diet with formula and reached full MOM by the second week of life. This represents the opportunity to address challenges of milk supply of mothers with premature infants in the NICU in the first two weeks after birth.

## 1. Introduction

Human breast milk is the gold standard for infant nutrition due to its numerous beneficial effects on infant and maternal health [1]. The World Health Organization (WHO) recommends that mother’s own milk (MOM) should be given to all preterm and low-birth-weight infants and that pasteurized donor milk (PDM) should be considered when MOM is not available [2]. Studies have shown that higher volumes of MOM are associated with improved neurodevelopmental outcomes [3] in premature infants and a decrease in the incidence and severity of prematurity-related morbidities like necrotizing enterocolitis [4,5,6,7], retinopathy of prematurity [7], late-onset sepsis [5], and bronchopulmonary dysplasia [8,9,10,11]. For a mother to supply a premature infant with enough breast milk, they must express their milk for an extended period of time before transitioning to direct breastfeeding. However, mother–infant separation, stress of neonatal intensive care unit (NICU) stays, maternal medical condition, and inadequate pumping may lead to insufficient milk supply, subsequent supplementation with PDB, and eventually formula [12,13,14,15,16,17,18].

Many studies have examined the relationship between infant diet and health outcomes [3,4,5,6,7,8,9,10,11]. However, assessing very-preterm infants’ feeding patterns during NICU hospitalization can be complex due to the use of several base milk types (MOM, PDM, and formula) and prolonged NICU stays. While several studies have quantified breast milk consumption during NICU stays [3,10,11,19,20], there are limited investigations of daily premature infant feeding trends, including both volume and milk type, as well as reports with precise cumulative feeding volume during the entire NICU hospitalization. Understanding the patterns of MOM intake in premature infants during NICU hospitalization will allow opportunities to improve mother’s milk supply. The purpose of this study was to quantify the type and total volume of daily milk intake in premature infants in the NICU from birth to 36^6/7^ weeks postmenstrual age (PMA).

## 2. Materials and Methods

This retrospective observational cohort study includes all inborn infants admitted to our AAP Level IV NICU at <33 weeks gestational age from January 2014 to December 2017. During the study period, our hospital was working towards Baby-Friendly Designation and many of the components of the WHO’s 10 Steps to Successful Breastfeeding relating to staff education had already been established. A standardized feeding advancement guideline focusing on the use of expressed human milk including MOM and PDM had been in place since 2009 and was revised in 2014 (Supplemental Table S1). Our guideline recommends starting colostrum administration within the first hours of life, trophic feeds with expressed human milk starting on day-of-life (DOL) one, advancing feeding volume and adding fortification based on birth gestational age (GA) and/or birth weight. When there was not enough MOM, after obtaining verbal consent from the parents, our guideline recommends PDM supplementation for infants <33 weeks PMA and formula supplementation for infants ≥34 weeks PMA. Since our median postmenstrual age at discharge was 36^6/7^, data were collected daily from birth to 36^6/7^ weeks PMA or discharge from the NICU, whichever occurred first. Data were not collected from infants who had contraindications for receiving MOM (maternal substance use, maternal medications contraindicated in breastfeeding, maternal HIV-positive status, maternal death), died during NICU hospitalization, or transferred to another facility before 36^6/7^ weeks PMA.

Study infants were categorized into two groups, exclusive human milk diet (EHM) and mixed diet depending on base milk intake during the entire hospitalization: MOM, PDM, and formula. The EHM diet group was defined as infants whose base milk type was MOM with or without PDM supplementation. The mixed diet group received a combination of formula and MOM and/or PDM.

Maternal demographics and risk factors (maternal age, race, ethnicity, antenatal steroids, pregnancy-induced hypertension, cesarean section, estimated blood loss, parity), neonatal demographics (birth gestational age, birth weight, sex), neonatal morbidities (late-onset sepsis, chronic lung disease, necrotizing enterocolitis, severe retinopathy of prematurity, severe intraventricular hemorrhage, survival without major morbidity), anthropometric measurements, and length of stay were collected from our NICU database.

Volume and type of base milk intake of every feed were collected from the electronic medical record (EMR). Percent volume of each feeding type was calculated for each day. Cumulative volume and percent of MOM intake were calculated. Average MOM %, PDM %, and formula % were summarized by day of life and PMA. Infants clinically made NPO (withholding feeds) and received 0 mL of base milk intake on a given day were excluded from analysis for that day. Other feeding variables including time to first colostrum (age at which infant received first maternal colostrum), PMA at first breastfeeding session, percent of infants discharged on any mother’s own milk, PMA at discharge, number of NPO days before full enteral feeds (150 mL/kg/day), and days to regaining birth weight were collected retrospectively from the EMR. Growth velocity was assessed for weight, occipitofrontal head circumference (OFC), and length. Weight growth velocity from birth to 36^6/7^ weeks PMA (or discharge) was calculated using Patel et al.’s 2-point exponential model [8]. Weekly OFC velocity and length growth velocity were also calculated (cm/wk).

Categorical data were compared between the EHM group and mixed diet groups using Chi-squared test. Continuous data were compared using Student *t*-test or Mann–Whitney rank sum test as appropriate. Feeding and neonatal outcomes were analyzed using univariable and multivariable quantile and logistic regression models with robust standard errors adjusting for gestational age and cesarean section. Statistical analysis was performed using STATA 14.0 (Statacorp, College Station, TX, USA). A *p* value < 0.05 was considered significant.

## 3. Results

**Figure 1:**
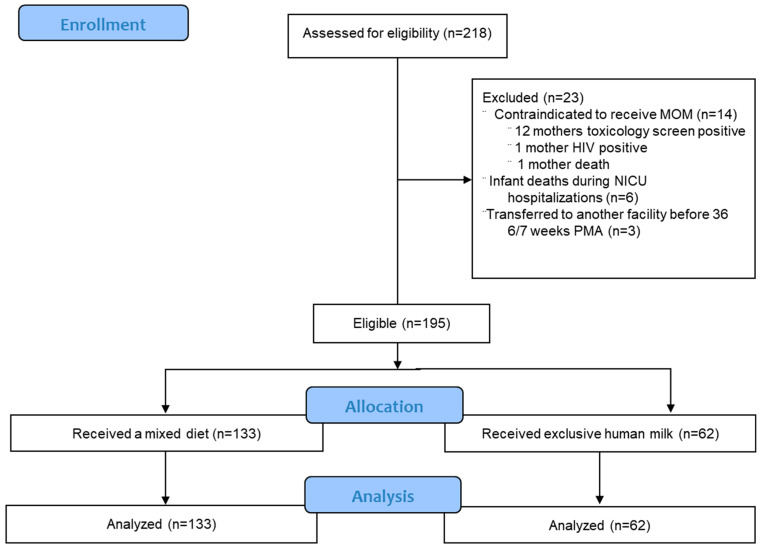
Flow chart for inclusion of participants. Total of 195 infants were eligible for the study.

During our study period, 218 premature infants were born, 23 of whom were excluded from our study. Therefore, 195 premature infants met eligibility requirements and were included in this study (Figure 1).

Of the 195 eligible infants, 133 (68%) received a mixed diet and 62 (32%) received an EHM diet. Fifty-eight percent (114/195) of infants were discharged by 36^6/7^ PMA. There were no significant differences in neonatal and maternal demographics except for birth gestational age, percent of cesarean section, and estimated blood volume loss between the two groups (Table 1). Estimated blood loss was lower in the EHM group but was not significant after adjusting for cesarean section percentage. In total, 98 percent of all infants received at least one feed of MOM and the median percentage of cumulative feeds as MOM was 77%. Cumulative volume and percent of MOM intake were greater in the EHM group.

**Figure 2:**
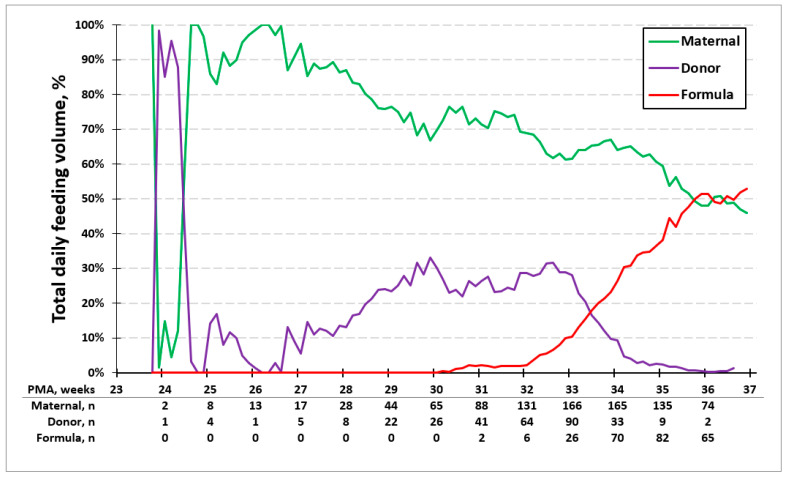
Average percentage of feeding type. Average percent volume of milk type each day by PMA. Between 32 and 34 weeks PMA, there is a switch from PDM to formula in the mixed diet group.

Figure 2 shows the average percent volume of base milk consumed each day for all study infants presented by PMA. There is a switch from PDM to formula in the mixed diet group between 32 and 34 weeks PMA per our feeding guideline. In the entire cohort, the daily percentage of MOM declines over time to ~50% at the time of discharge.

**Figure 3:**
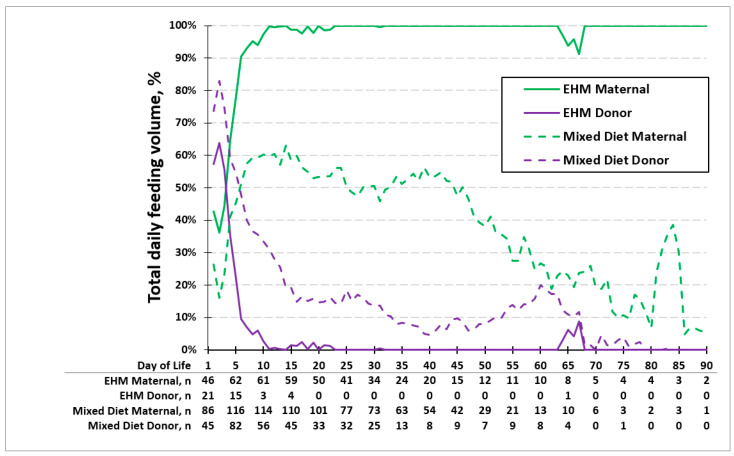
Average percentage of feeding type in exclusive human milk (EHM) group and mixed diet group. Age at which 50% of the base milk was MOM in the EHM group was on day 4 and day 6 in the mixed diet group. The maximum percentage of MOM was 100% in the exclusive human milk group and 63% in the mixed diet group by 2 weeks of life.

Figure 3 shows the average percent volume of MOM and PDM consumed each day of life in the EHM group and the mixed diet group. The age at which 50% of the base milk was MOM in the EHM group was earlier (day 4) compared to the mixed diet group (day 6). In the EHM group, infants received 100% of their feeds as MOM by 2 weeks of life. The use of donor milk between 65 and 70 days was due to one mother being sick and unavailable to visit the NICU to provide MOM. In the mixed diet group, the maximum average daily percentage of MOM received was 63% by 2 weeks of life, with a slow decline thereafter to less than 10%.

Feeding outcomes are presented in Table 2. Time to first colostrum in the EHM group was earlier than the mixed diet group after adjusting for gestational age and cesarean section. The percent of infants discharged on any MOM was more in the EHM group compared to the mixed diet group. There was no significant difference in the other feeding outcomes or growth parameters.

Neonatal outcomes and morbidities are presented in Table 3. PMA at discharge and length of stay was lower in the EHM group but not significant after adjusting for birth gestational age and cesarean section. Even though length of stay does not reach statistical difference after adjusting for birth gestational age and cesarean section, the direction may have clinical significance with the EHM group having a shorter length of stay than the mixed diet group. There was no difference in neonatal unadjusted morbidities between the two groups or after adjusting for the baseline differences in birth gestational age and cesarean section.

## 4. Discussion

In our retrospective observation cohort study, more than half of our total population consumed MOM as their primary milk source during NICU hospitalization and the majority had MOM included in their diet at the time of hospital discharge. The EHM group was able to transition to exclusive MOM by 2 weeks of life. However, the mixed diet group had a maximum provision of MOM of 63% by 2 weeks of life, which declined with time.

Our feeding practice of early colostrum administration and feeding advancement is similar to other recommendations and practices [21,22]. The literature quantifying milk type, daily feeding trends, and percentage of MOM throughout the entire NICU admission is limited [3,10,11,19,20]. Our study included a daily data collection of every infant feed from birth through 36^6/7^ weeks PMA. This complete dataset allowed us to have a comprehensive understanding of premature infant feeding patterns throughout the NICU hospitalization. In our study, 98% of infants received at least one feeding with MOM and a median of 77% total feeding volume as MOM. This is similar to a multicenter randomized study that reported a median of approximately 75% of mother’s own milk intake across their study groups [4]. A multicenter Italian study reported that 31% of preterm infants received an EHM diet during the last 72 h prior to NICU discharge [19]. Cabrera et al. [20] reported that the majority of infants received partial mother’s own milk with only 3% of infants receiving exclusive maternal milk in a hospital without PDM. In our study, 32% of our population was fed an EHM diet during the entire hospitalization (or through 36^6/7^ weeks PMA). The median length of stay was 32 days in the NICU, which is a challenging duration to supply an adequate amount of mother’s own milk given the stressors associated with having an infant in the NICU (e.g., maternal–infant separation, maternal stress, prolonged exclusive pumping) [23,24,25].

Early colostrum expression has been linked to increased maternal milk production [26]. In our study, earlier initiation of colostrum feeding was associated with a higher total percentage of MOM intake in the EHM group. Cesarean delivery is a known risk factor for low milk volumes during the first weeks of life [27]. Time to the first colostrum feed was earlier in the EHM group after adjusting for gestational age and cesarean delivery.

Our study showed that the EHM group reached 100% of feeds as MOM by 2 weeks of life. In contrast, the mixed diet group only had a maximum daily percentage of 63% MOM by 2 weeks of life and never reached 100% MOM. Previous studies have shown that mothers who accomplish coming to volume (CTV), defined as producing >500 mL/day of MOM by 2 weeks post-delivery, are more likely to provide MOM throughout the NICU stay [28]. Clinically, the percentage of MOM feedings is another way to assess mothers’ milk supply; the mothers of infants with a mixed diet had difficulty reaching CTV. In very-low-birth-weight (VLBW) infants, the maternal milk supply can be less than the CTV and still provide exclusive maternal milk for the infant; for example, a 1.5 kg baby at full enteral feeds of 160 mL/kg/day would only need 240 mL/day of MOM (half of CTV of 500 mL/day). Hence, if an infant is not receiving 100% MOM by 2 weeks, those mothers have not achieved CTV. While this could be due to multiple factors, including fewer pumping sessions, our study highlighted the importance of reaching CTV within the first 2 weeks to provide an EHM diet. A targeted approach to supporting mothers to achieve earlier colostrum expression and more pumping sessions within the first 2 weeks post-delivery could improve CTV [29,30].

The literature has suggested that higher volumes of MOM decrease prematurity-related morbidities [4,5,7,8]. We did not observe a difference in the neonatal morbidities between the two groups. This is likely due to the overall high MOM intake in our cohort. Also, our study may not be powered to detect differences in these outcomes.

The main strength of our study is that we collected daily feeding data from birth through 36^6/7^ weeks PMA or discharge. This dataset captures daily feeding types and accurately describes the cumulative MOM volume during the entire NICU hospitalization. This retrospective observation study is from a single-center cohort with high MOM initiation rates and unlimited access to PDM, which may limit the generalizability to like centers.

The key steps to achieving exclusive human milk feeding in premature infants are initiating early colostrum feedings and reaching full MOM feedings by 2 weeks of life. Increasing the provision of MOM by providing intensive lactation support for mothers in the first 2 weeks after delivery should be promoted in all neonatal intensive care units.

## Figures and Tables

**Table 1:** Neonatal and maternal demographics of participants and cumulative MOM intake of each group.

	Mixed Diet *n* = 133	Exclusive Human Milk *n* = 62	*p* Value *
Birth Gestational Age, weeks	30.9 (28.7–31.9)	31.7 (29.6–32.3)	0.03
Birth Weight, grams	1420 (1140–1830)	1634 (1250–1931)	0.1
Male Gender, %	62	50	0.1
Maternal Age, years	29 (24–35)	31 (27–34)	0.3
Parity	1 (0–2)	1 (0–2)	0.09
Maternal Race			0.9
Asian, %	10	10	
Black, %	3	3	
White, %	85	85	
Other, %	2	2	
Maternal Hispanic Ethnicity, %	77	73	0.5
Antenatal Steroids, %	95	95	0.9
Maternal Pre-Eclampsia, %	24	16	0.2
Cesarean Section, %	67	50	0.02
Estimated Blood Loss	800 (400–1000)	550 (200–800)	0.01 (0.1) ^#^
Cumulative MOM Intake, mL	4192 (1620–7614)	7209 (5001–10,754)	<0.001
Cumulative % MOM Intake	49 (24–78)	99 (96–100)	<0.001

Descriptive statistics are presented as % or median (IQR). * *p* values comparing mixed diet and exclusive human milk groups using Chi-squared test for categorical data and Student *t*-test or Mann–Whitney rank sum test as appropriate for continuous data. ^#^ *p* value after adjusting for cesarean section.

**Table 2:** Feeding outcomes and growth.

	Mixed Diet *n* = 133	Exclusive Human Milk *n* = 62	Correlation Coefficient/Odds Ratio (95% CI)	*p* Value *	Correlation Coefficient/Odds Ratio (95% CI)	Adjusted *p* Value ^@^
Time to First Colostrum, hour	4.9 (2.7–12.6)	3.1 (1.4–8.0)	−1.9 (−4.1, 0.3)	0.091	−2.5 (−4.4, −0.5)	0.013
PMA First Breastfeed, weeks	32.9 (32.3–33.4)	32.7 (32.1–33.1)	−0.2 (−0.5, 0.2)	0.351	0.0 (−0.4, 0.3)	0.85
NPO, days	0 (0–1)	0 (0–1)	0.0 (−0.5, 0.5)	1	0.1 (−0.1, 0.2)	0.565
MOM at Discharge, % ^§^	78	100	NA	<0.001	NA	NA
Regaining Birth Weight, days	10 (7–12)	9 (8–11)	0.0 (−1.2, 1.2)	0.967	0.0 (−1.2, 1.2)	0.971
Weight Growth Velocity, g/kg/d	13.5 (10.9–15.0)	11.7 (9.5–14.9)	−1.3 (−2.7, 0.0)	0.054	−0.6 (−1.6, 0.5)	0.277
OFC Growth Velocity, cm/wk	0.78 (0.63–0.89)	0.73 (0.54–0.88)	0.0 (−0.1, 0.1)	0.59	0.0 (−0.1, 0.1)	0.643
Length Growth Velocity, cm/wk	0.98 (0.80–1.12)	0.92 (0.70–1.12)	0.0 (−0.2, 0.1)	0.586	0.0 (−0.1, 0.1)	0.889

Descriptive statistics are presented as % or median (IQR). OFC = occipitofrontal circumference. * *p* values comparing mixed diet and exclusive human milk groups using Chi-squared test for categorical data and Student *t*-test or Mann–Whitney rank sum test as appropriate for continuous data. ^@^ Adjusted for gestational age and cesarean section. ^§^ Chi-squared test was performed instead of a regression model due to the lack of events in one group.

**Table 3:** Neonatal outcomes and morbidities.

	Mixed Diet *n* = 133	Exclusive Human Milk *n* = 62	Correlation Coefficient/Odds Ratio (95% CI)	*p* Value *	Correlation Coefficient/Odds Ratio (95% CI)	Adjusted *p* Value ^@^
PMA at Discharge, weeks	36.6 (35.6–38.0)	35.6 (35.0–38.0)	−1.0 (−1.9, −0.1)	0.03	−0.6 (−1.3, 0.2)	0.1
Length of Stay, days	44 (26–64)	32 (21–50)	−12 (−22, −2)	0.02	−9 (−18, 0)	0.052
Necrotizing Enterocolitis, %	0.8	1.6	2.2 (0.1, 35.2)	0.6	2.4 (0.1, 59.9)	0.6
Severe ROP, %	3.8	4.8	1.3 (0.3, 5.6)	0.7	1.3 (0.3, 5.1)	0.7
Late-Onset Sepsis, % ^#^	4.5	0	NA	0.2	NA	NA
Chronic Lung Disease, %	15.8	12.9	0.8 (0.3, 1.9)	0.6	1.0 (0.4, 2.8)	1
Severe Intraventricular Hemorrhage, %	3.8	1.6	0.4 (0.0, 3.7)	0.4	0.4 (0.0, 4.2)	0.4
Survival w/o Major Morbidity, %	78.2	87.1	1.9 (0.8, 4.4)	0.1	1.7 (0.7, 4.3)	0.2

Descriptive statistics are presented as % or median (IQR). PMA = postmenstrual age; ROP = retinopathy of prematurity. * *p* values comparing mixed diet and exclusive human milk groups using Chi-squared test for categorical data and Student *t*-test or Mann–Whitney rank sum test as appropriate for continuous data. ^@^ Adjusted for gestational age and cesarean section. ^#^ Chi-squared test performed instead of a regression model due to the lack of events in one group.

## Data Availability

Dataset is available on request from the authors.

## Supplementary Materials

The following supporting information can be downloaded at: https://www.mdpi.com/article/10.3390/children12040431/s1. Table S1: Feeding Guidelines.
