# Supplementary material for: Type and Volume of Milk Intake in Premature Infants <33 Weeks Gestational Age in the Neonatal Intensive Care Unit
Source: Children (Basel). 2025 Mar 29;12(4):431. doi: 10.3390/children12040431 (PMC12025542; doi:10.3390/children12040431)
Supplement: Supplementary file 1 [file children-12-00431-s001.zip › children-3533620-supplementary.pdf]

SANTA CLARA VALLEY HEALTHCARE

GI – SCVMC Premie Feeding Guidelines Revised

Day of Feeding	Type of feed	BW/GA 401 – 700 gm 23-25wks ml/kg/day	BW/GA 701 – 1000 gm 26-28wks ml/kg/day	BW/GA 1001 – 1250 gm 29-30wks ml/kg/day	BW/GA 1250 – 1500 gm 31-32wks ml/kg/day	Date/reason if not advanced per protocol
1	EHM	**15**	**15**	**20**	**20**	
2	EHM	**15**	**15**	**20**	**40**	
3	EHM	**15**	**15**	**40**	**60**	
4	EHM	**15**	**30**	**60**	**80**	
5	EHM	**15**	**45**	**80**	**100**<br>**Conc. TPN 100/kg**	
6	EHM	**30**	**60 + Prolalcta 4**	**100**<br>**Conc. TPN 100/kg**	**120 + HMF**	
7	EHM	**45**	**80**	**120 + HMF**	**140**	
8	EHM	**60 + Prolacta4**	**100**<br>**Conc. TPN 100/kg**	**140**	160	
9	EHM	**80**	**120**<br>**Prolacta 6**	160	180	
10	EHM	**100**<br>**Conc. TPN 100/kg**	140	180		
11	EHM	**120**<br>**Prolacta 6**	160			
12	EHM	140	180			
13	EHM	160				

12.4.14
